# Combined bowel urgency, clinical outcomes, and quality of life improvement in mirikizumab-treated ulcerative colitis: a step toward comprehensive disease management

**DOI:** 10.1093/crocol/otag051

**Published:** 2026-06-10

**Authors:** Stefan Schreiber, Jianmin Wu, Yajie Duan, Anthony Keohane, Seyedehsan Navabi, David T Rubin

**Affiliations:** Department of Internal Medicine I, University Hospital Schleswig-Holstein, Kiel, Germany; Eli Lilly and Company, Indianapolis, IN, United States; Eli Lilly and Company, Indianapolis, IN, United States; HaaPACS GmbH, StatisticsSchriesheim, Germany, Europe; United Medical Doctors, Murrieta, CA, United States; University of Chicago Medicine Inflammatory Bowel Disease Center, Chicago, IL, United States

**Keywords:** bowel urgency, combined clinical and patient-reported outcomes, IBDQ, mirikizumab, ulcerative colitis

## Abstract

**Background:**

With increasing emphasis on patient-centric care, treatment goals for ulcerative colitis (UC) now include bowel urgency (BU), stool frequency, and rectal bleeding (RB) resolution, enhancement of overall well-being, and endoscopic remission (ER). This analysis evaluated the impact of adding BU improvement to clinical remission, defined by the modified Mayo score (RB, stool frequency, ER), on quality of life assessed by the Inflammatory Bowel Disease Questionnaire (IBDQ). The effect of mirikizumab on achieving combined clinical, BU, and IBDQ remissions was also evaluated in the LUCENT-1 and -2 trials.

**Methods:**

In LUCENT-1, patients were randomized 3:1 (mirikizumab to placebo) every 4 weeks. At Week 12, mirikizumab responders were re-randomized 2:1 (mirikizumab to placebo) for 40 additional weeks in LUCENT-2. BU improvement was assessed using clinically meaningful improvement (Urgency Numeric Rating Scale [UNRS] ≥3-point change) and BU remission (UNRS = 0 or 1). The impact of mirikizumab on achieving single and combined outcomes was determined at Weeks 12 and 52.

**Results:**

Significant IBDQ improvement at both timepoints (*P ≤ *.01) was observed by achieving both BU improvement and clinical remission versus clinical remission alone. Significantly more mirikizumab-treated patients achieved single and combined endpoints of clinical, BU, and IBDQ remissions versus placebo (*P ≤ *.05).

**Conclusions:**

The addition of BU improvement in patients who had already attained clinical response or remission is associated with greater quality of life. Mirikizumab delivered comprehensive benefit for patients with UC by relieving BU symptoms, resolving endoscopic inflammation, and enhancing overall well-being concurrently.

## Introduction

Ulcerative colitis (UC) is a challenging inflammatory bowel disease with symptoms including rectal bleeding, increased stool frequency, and bowel urgency (BU), the sudden urge for bowel evacuation.^1^ Current treatment strategies primarily focus on remission of rectal bleeding, normalization of stool frequency, and endoscopic healing.^2^ However, these goals may overlook the complexities of patients’ experiences, including BU, a disruptive symptom that profoundly affects quality of life and psychosocial functioning and can be distressing even after the patient achieves clinical remission.^3^

BU is distinct from frequent stools and is often underestimated by healthcare providers.^4^ Patients may also refrain from discussing BU due to embarrassment, despite it being one of the symptoms they most desire to improve.^5^^,^^6^ Due to these issues and lack of awareness or understanding of the importance of evaluating BU, healthcare providers may not adequately address the symptom. The Urgency Numeric Rating Scale (UNRS), a validated, patient-centric tool, was developed to address this healthcare gap by assessing BU severity.^7^^,^^8^ UNRS has been adopted in UC clinical research^9^^,^^10^ and can facilitate better assessment of treatment outcomes by incorporating patient experience and ultimately improving the quality of UC management.

Although BU has not been included in most UC disease activity indices such as the modified Mayo score (mMS), BU remission and clinically meaningful improvement (CMI) are associated with better clinical outcomes, such as clinical remission, endoscopic remission, and quality of life.^11^ The American College of Gastroenterology guidelines include resolution of BU as a goal and define UC remission as no urgency, no rectal bleeding, normal stool frequency, and endoscopic remission or mucosal healing (Mayo endoscopic subscore of  ≤ 1).^12^^,^^13^

Mirikizumab, an anti-interleukin-23p19 IgG4 monoclonal antibody, demonstrated efficacy and favorable safety in patients with moderately-to-severely active UC in the Phase 3 LUCENT-1 induction (NCT03518086) and LUCENT-2 maintenance (NCT03524092) trials.^9^^,^^11^ This exploratory analysis aimed to evaluate the association of adding BU improvement to clinical response or remission defined by mMS, on quality of life as assessed by the Inflammatory Bowel Disease Questionnaire (IBDQ) in patients from LUCENT-1 and LUCENT-2. It also assessed the impact of mirikizumab on achieving the combined endpoints of clinical response or remission, BU remission, and IBDQ remission.

## Materials and methods

### Patients and study design

**Figure 1:**
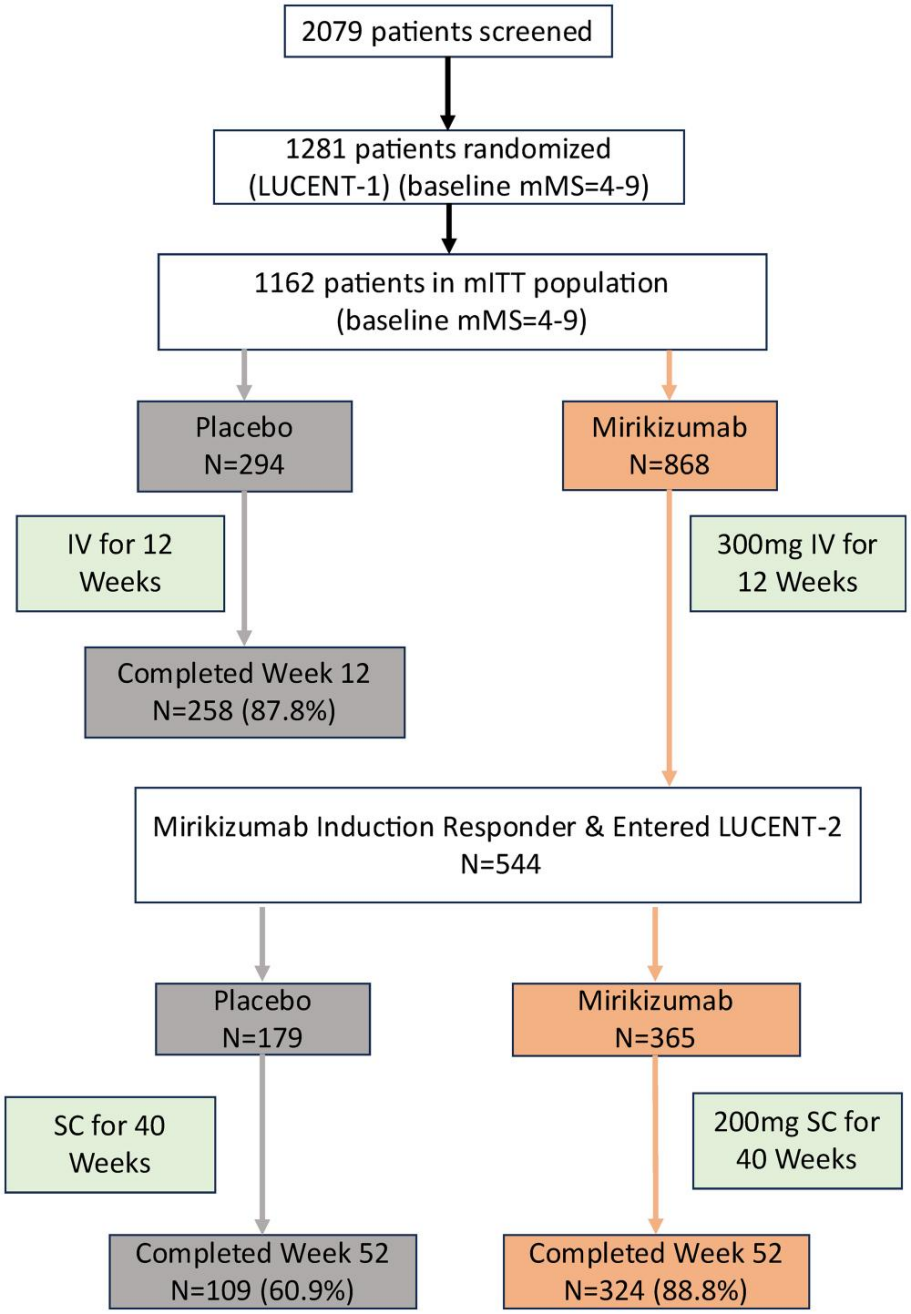
LUCENT-1 and LUCENT-2 trial patient disposition. IV, intravenously; mITT, modified intention-to-treat; mMS, modified Mayo score; SC, subcutaneously.

Patient disposition for the LUCENT trials has been reported previously.^9^ A summary flowchart for the LUCENT-1 and -2 trials is in Figure 1. Key clinical study methods are noted along with details for the current analyses. Detailed methods and baseline characteristics for the LUCENT-1 induction (NCT03518086) and LUCENT-2 maintenance (NCT03524092) studies have been previously described by D’Haens et al.^9^ LUCENT-1 was a 12-week induction trial followed by LUCENT-2, a 40-week randomization, withdrawal, and maintenance trial. Among the primary efficacy population in LUCENT-1, 33.0% from the placebo group and 37.4% from the mirikizumab-treated group had failed at least one anti-TNF therapy. Specifically, 20.1% (placebo) and 18.3% (mirikizumab-treated) had failed vedolizumab, and 2.0% (placebo) and 3.9% (mirikizumab-treated) had failed tofacitinib. Additionally, 40.1% (placebo) and 41.6% (mirikizumab-treated) had failed at least one biologic or tofacitinib. It should be noted that weeks reported are cumulative; eg, week (W) 40 of LUCENT-2 refers to W52 of continuous treatment and baseline refers to LUCENT-1 W0.

### Study assessments

As previously described, clinical remission was the primary endpoint for this analysis while BU CMI, BU remission, clinical response, IBDQ response, and IBDQ remission were secondary endpoints.^9^ Assessment of disease activity utilized the mMS. Clinical remission was defined as stool frequency subscore of 0 or 1 with a ≥ 1-point decrease from induction baseline, rectal bleeding subscore of 0, and endoscopic subscore (ES) = 0 or ES = 1 (excluding friability). Clinical response was defined as a decrease in the mMS of ≥2 points and ≥30% decrease from baseline, and a decrease of ≥1 point in the rectal bleeding subscore from baseline or a rectal bleeding score of 0 or 1.

The UNRS is a single item patient-reported outcome that measures severity of urgency to have a bowel movement (sudden or immediate need) in the past 24 hours using an 11-point numeric rating scale ranging from 0 (no urgency) to 10 (worst possible urgency). Patients were provided with an electronic diary tool during screening to record information daily pertaining to their severity of urgency. BU CMI was defined as a ≥ 3-point change in UNRS and BU remission was defined as a UNRS score of 0 or 1, both measured in patients with a baseline UNRS of ≥3.^11^

The IBDQ is a 32-item patient-completed questionnaire that measures 4 domains of patients’ lives: symptoms directly related to the primary bowel disturbance, systemic symptoms, emotional function, and social function.^14^^,^^15^ Responses are graded on a 7-point Likert scale in which 1 denotes “a very severe problem” and 7 denotes “not a problem at all.” Scores range from 32 to 224 with a higher score indicating a better quality of life. Patients recorded their responses to the IBDQ electronically as source data in the tablet device at appropriate visits. A summary of definitions for studied outcomes can be found in Table 1.

**Table 1:** Definitions of studied outcomes.

Outcome	Definition
**BU CMI**	≥3-point change, among patients with baseline UNRS ≥3
**BU remission**	UNRS score of 0 or 1, among patients with baseline UNRS ≥3
**Clinical response**	Decrease of ≥2 points and ≥30% from baseline in the mMS, plus a rectal bleeding subscore of 0 or 1 or a decrease of ≥1 point from baseline
**Clinical remission**	Stool frequency subscore of 0 or 1 with a ≥ 1-point decrease from baseline, rectal bleeding subscore of 0, and ES = 0 or ES = 1 (excluding friability)
**IBDQ response**	≥16-point improvement from baseline in IBDQ total score
**IBDQ remission**	IBDQ total score ≥170

Abbreviations: BU, bowel urgency; CMI, clinically meaningful improvement; ES, endoscopic subscore; IBDQ, Inflammatory Bowel Disease Questionnaire; mMS, modified Mayo score; UNRS, Urgency Numeric Rating Scale.

### Statistical analyses

The analysis population for LUCENT-1 outcomes was the modified intent-to-treat population, which included all randomized patients who received any amount of study treatment. Mirikizumab W12 induction responders who were re-randomized to mirikizumab or placebo in LUCENT-2 were analyzed for outcomes at W52. Unless otherwise specified, baseline values for analyses in LUCENT-2 refer to the values collected at LUCENT-1 baseline.

Among all patients (pooled from mirikizumab and placebo groups) with baseline UNRS ≥3 who achieved clinical response at W12 or W52, the association between BU improvement (CMI or remission) and IBDQ (response or remission) was evaluated using the chi-square test of association. The association analysis was repeated among patients who achieved clinical remission at W12 and W52. Additionally, the association analyses were also performed within the mirikizumab-treated and placebo-treated patients separately. The effect of mirikizumab versus placebo on individual and combined endpoints was assessed in patients with UNRS ≥3 at baseline of LUCENT-1 using Cochran-Mantel-Haenszel tests. Endpoints at W12 and W52 included clinical response and remission based on the mMS, BU CMI, BU remission, IBDQ remission, and combinations of two or three of these endpoints. For missing data, non-responder imputation was used for binary variables; modified baseline observation carried forward was used for continuous variables. *P-*values were calculated using the Cochran-Mantel-Haenszel test adjusted by prior biologic or tofacitinib failure (yes/no), prior baseline corticosteroid use (yes/no), region (North America/Europe/other), and baseline disease activity (LUCENT-1 only; mMS: [4-6] or [7-9]).

### Ethical considerations

All patients were required to provide written informed consent for participation in the studies. The protocol, amendments, and consent documentation were approved by local ethical review boards. The study was registered at the European Network of Centers for Pharmacoepidemiology and Pharmacovigilance and was conducted according to consensus ethical principles derived from international guidelines, including the Declaration of Helsinki and Council for International Organizations of Medical Sciences International Ethical Guidelines, applicable International Council for Harmonization Good Clinical Practice Guidelines, and applicable laws and regulations.

## Results

### Improved IBDQ outcomes associated with the addition of BU CMI to clinical response or remission

**Figure 2:**
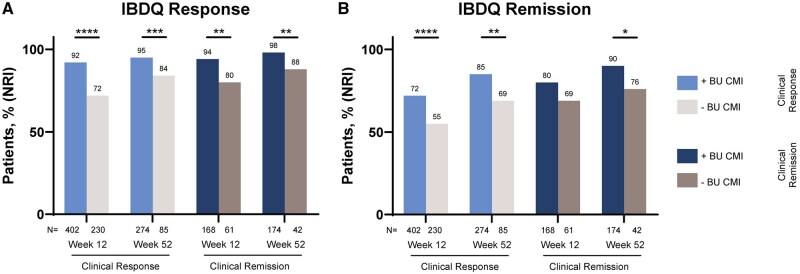
IBDQ response and remission with and without BU CMI at Weeks 12 and 52. The addition of BU CMI to clinical response/remission is associated with higher IBDQ response at Weeks 12 and 52 (A). The addition of BU CMI to clinical response/remission is associated with higher IBDQ remission at Weeks 12 and 52 (B). *P ≤.05; **P ≤.01; ***P ≤.001; ****P ≤.0001, with (+) BU CMI vs without (−) BU CMI, chi-square test of association. The difference between with and without BU CMI was not adjusted for covariates and was not continuity corrected. Patients pooled from LUCENT mirikizumab + groups with baseline Urgency Numeric Rating Scale score ≥3 and clinical response or clinical remission at Week 12 or 52. BU, bowel urgency; CMI, clinically meaningful improvement; IBDQ, Inflammatory Bowel Disease Questionnaire; *N*, number of patients in the denominator; NRI, non-responder imputation.

Patients’ baseline demographics and disease characteristics in LUCENT-1 and LUCENT-2 have been previously reported.^9^^,^^11^ When adding BU CMI to the outcome of clinical response at W52, significantly more patients achieved IBDQ response (95% vs 84%, *P* ≤ .001, Figure 2A) and IBDQ remission (85% vs 69%, *P* ≤ .01, Figure 2B) compared with those without BU CMI. Additionally, when adding BU CMI to the outcome of clinical remission at W52, significantly more patients achieved IBDQ response (98% vs 88%, *P* ≤ .01, Figure 2A) and IBDQ remission (90% vs 76%, *P* ≤ .05, Figure 2B) compared with those without BU CMI. A similar trend was observed at W12, although the effect on IBDQ remission rate of adding BU CMI to clinical remission was not statistically significant.

### Improved IBDQ outcomes associated with the addition of BU remission to clinical response or remission

**Figure 3:**
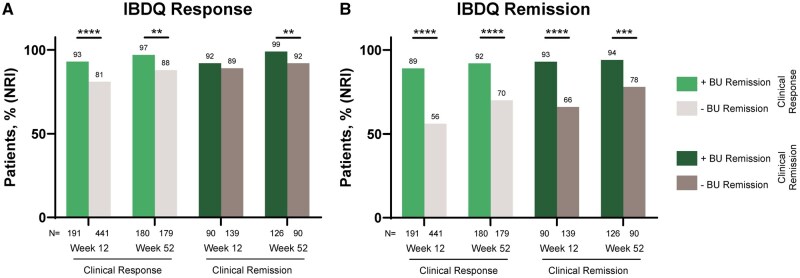
IBDQ response and remission with and without BU remission at Weeks 12 and 52. The addition of achieving BU remission to clinical response**/**remission is associated with higher IBDQ response at Weeks 12 and 52 (A). The addition of BU remission to clinical response/remission is associated with higher IBDQ remission at Weeks 12 and 52 (B). **P ≤.01; ***P ≤.001; ****P ≤.0001, with (+) BU remission vs without (−) BU remission, chi-square test of association. The difference between with and without BU remission was not adjusted for covariates and was not continuity corrected. Patients pooled from LUCENT mirikizumab + groups with baseline Urgency Numeric Rating Scale score ≥3 and clinical response or clinical remission at Week 12 or 52. BU, bowel urgency; CMI, clinically meaningful improvement; IBDQ, Inflammatory Bowel Disease Questionnaire; *N*, number of patients in the denominator; NRI, non-responder imputation.

When adding BU remission, defined as a UNRS score of 0 or 1, to the outcomes of clinical response and clinical remission at W52, significantly more patients achieved IBDQ response compared with those without BU remission (clinical response with vs without BU remission: 97% vs 88%, *P* ≤ .01, clinical remission with vs without BU remission: 99% vs 92%, *P* ≤ .01, Figure 3A). Similarly, about 92% of patients achieved IBDQ remission if they achieved both clinical response and BU remission vs 70% of patients achieved clinical response without BU remission simultaneously (*P* ≤ .0001). About 94% of patients achieved IBDQ remission if they achieved both clinical remission and BU remission vs 78% of patients who achieved clinical remission without BU remission simultaneously (*P* ≤ .001) (Figure 3B). A similar trend was observed at W12, although the effect on IBDQ response rate of adding BU remission to clinical remission was not statistically significant.

Figure S1 illustrates the association of BU improvement (BU CMI or BU remission) to IBDQ outcome at W12 and W52 by treatment groups, with a consistent association observed across both the mirikizumab and placebo subgroups.

### Significantly more mirikizumab-treated patients compared with placebo achieved combined endpoints, including clinical remission, BU remission, and IBDQ remission

**Figure 4:**
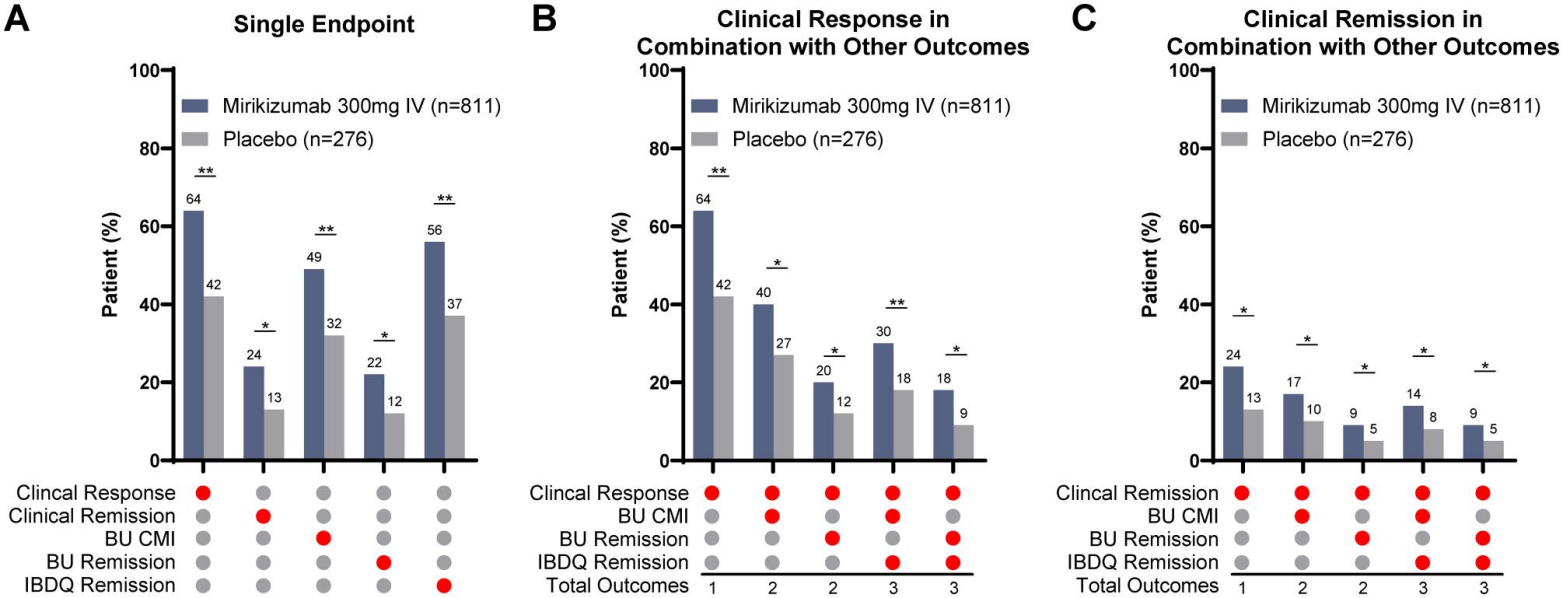
Proportion of patients achieving clinical response, clinical remission, BU CMI, BU remission, and IBDQ remission (A), clinical response (B), and clinical remission (C) in combination with other endpoints at Week 12 in the LUCENT-1 induction trial. *P≤.05; **P≤.0001. BU, bowel urgency; CMI, clinically meaningful improvement; IBDQ, Inflammatory Bowel Disease Questionnaire; IV, intravenously.

**Figure 5:**
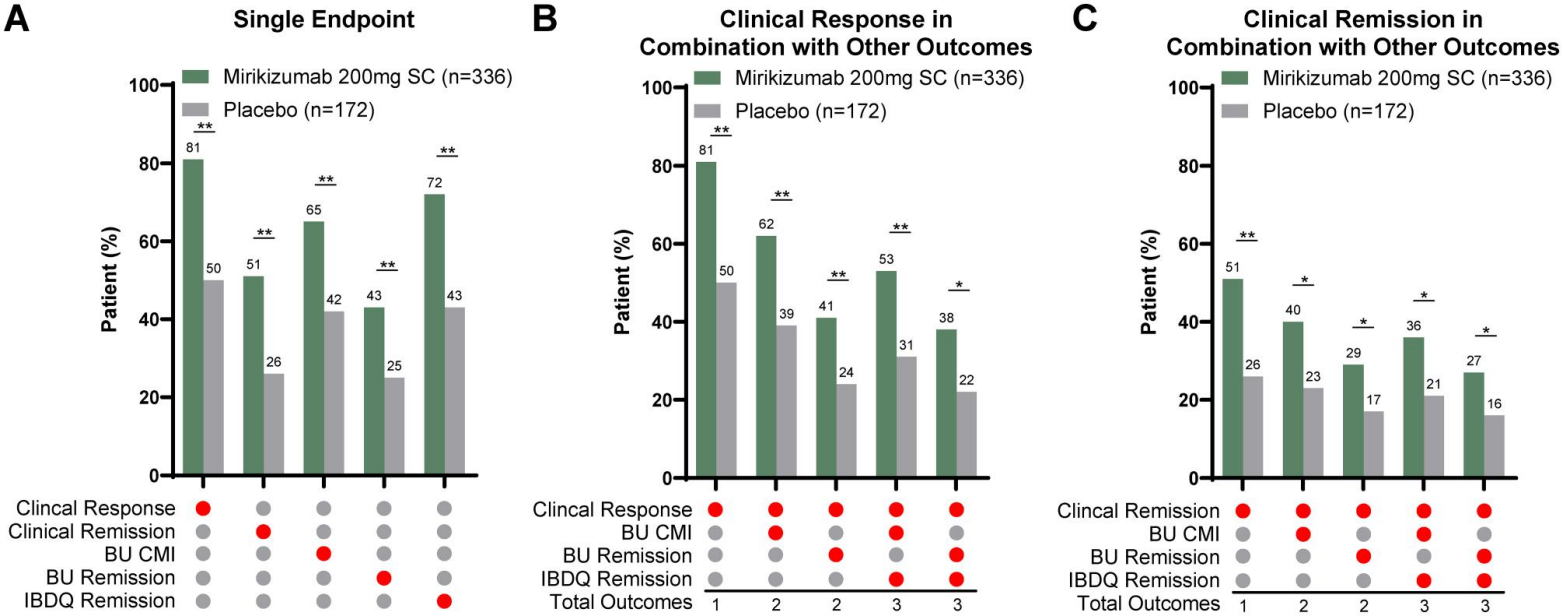
Proportion of patients achieving clinical response, clinical remission, BU CMI, BU remission, and IBDQ remission (A), clinical response (B), and clinical remission (C) in combination with other endpoints at Week 52 in the LUCENT-2 maintenance trial. *P≤.05; **P≤.0001. BU, bowel urgency; CMI, clinically meaningful improvement; IBDQ, Inflammatory Bowel Disease Questionnaire; SC, subcutaneously.

The impact of mirikizumab on achieving different targets, including clinical response, clinical remission, BU CMI, BU remission, and IBDQ remission, was assessed for both LUCENT induction and maintenance trials. At W12 (Figure 4, left) and W52 (Figure 5, left), significantly greater proportions of mirikizumab-treated versus placebo-treated patients achieved the individual endpoints clinical response, clinical remission, BU CMI, BU remission, and IBDQ remission (*P ≤ *.05 or *P ≤ *.0001 in all cases). When assessing the combined endpoints involving dual targets (clinical response/remission and BU CMI/BU remission), significantly greater proportions of patients treated with mirikizumab versus placebo achieved each dual endpoint, both at W12 (Figure 4, center), and W52 (Figure 5, center) (*P ≤ *.05 or *P ≤ *.0001 in all cases). Lastly, when assessing the combined endpoints involving several targets (clinical response/remission, BU CMI/BU remission, and IBDQ remission), greater proportions of patients treated with mirikizumab versus placebo achieved each triple endpoint at W12 (Figure 4, right) and W52 (Figure 5, right) (*P ≤ *.05 or *P ≤ *.0001 in all cases). Particularly, 27% of mirikizumab-treated patients achieved the triple endpoints (clinical remission, BU remission, and IBDQ remission) vs 16% of patients on placebo at Week 52 (*P ≤ *.0001).

### Demographics and baseline characteristics for patients achieving combined endpoints

A summary of demographics and other baseline characteristics for patients achieving versus not achieving combined endpoints of clinical remission, BU remission, and IBDQ remission can be found in Table 2 (LUCENT-2, Week 52 maintenance responders/non-responders) and Table S1 (LUCENT-1, Week 12 induction responders/non-responders). A higher prevalence of left-sided disease (mirikizumab: 71.4% vs 62.4%; placebo: 82.1% vs 62.5%), and biologic- and small molecule-naïve patients (mirikizumab: 70.3% vs 61.2%, placebo: 78.6% vs 60.4%) was observed among responders to combined endpoints than non-responders. Notably, among the mirikizumab-treated patients, responders had a higher proportion with severe endoscopic disease (ES = 3) (72.5% vs 64.5%) and a higher proportion with mMS score of 7-9 (61.5% vs 51.0%) compared with non-responders, whereas the placebo group showed the opposite trend.

**Table 2:** Summary of demographics and other baseline characteristics for mirikizumab- and placebo-treated patients achieving versus not achieving combined clinical remission, BU remission, and IBDQ remission at Week 52.

	Combined endpoint: clinical remission, BU remission, and IBDQ remission			
	Maintenance: Week 52 Mirikizumab 200 mg SC (*N* = 336)		Maintenance: Week 52 Placebo SC (*N* = 172)	
	Responders^a^ (*M* = 91)	Non-responders^b^ (*M* = 245)	Responders^a^ (*M* = 28)	Non-responders^b^ (*M* = 144)
**Age, mean years (SD)**	43.8 (14.8)	42.9 (13.7)	41.8 (14.5)	40.9 (12.3)
**Male, *n* (%)**	54 (59.3%)	141 (57.6%)	14 (50.0%)	87 (60.4%)
**BMI, *n* (%)**				
** Normal**	49 (53.8%)	126 (51.4%)	17 (60.7%)	77 (53.5%)
** Underweight**	4 (4.4%)	22 (9.0%)	2 (7.1%)	6 (4.2%)
** Overweight/obese/extremely obese**	38 (41.8%)	97 (39.6%)	9 (32.1%)	61 (42.4%)
**Disease location, *n* (%)**				
** Left-sided colitis**	65 (71.4%)	153 (62.4%)	23 (82.1%)	90 (62.5%)
** Pancolitis**	26 (28.6%)	89 (36.3%)	5 (17.9%)	53 (36.8%)
** Proctitis**	0	3 (1.2%)	0	1 (0.7%)
**mMS, *n* (%)**				
** [0-6]**	35 (38.5%)	120 (49.0%)	12 (42.9%)	59 (41.0%)
** [7-9]**	56 (61.5%)	125 (51.0%)	16 (57.1%)	85 (59.0%)
**Mayo endoscopic subscore (ES), *n* (%)**				
** Moderate disease (ES = 2)**	25 (27.5%)	87 (35.5%)	12 (42.9%)	55 (38.2%)
** Severe disease (ES = 3)**	66 (72.5%)	158 (64.5%)	16 (57.1%)	89 (61.8%)
**Bowel Urgency Severity (UNRS), mean (SD)**	6.1 (1.8)	6.5 (1.8)	6.4 (1.7)	6.4 (1.7)
**Fecal calprotectin, µg/g, median (Q1, Q3)**	1617.0 (561.0, 3276.0)	1482.0 (581.0, 2948.0)	1984.0 (823.0, 4054.0)	1766.5 (748.0, 3514.0)
**CRP, mg/L, median (Q1, Q3)**	4.0 (1.9, 8.5)	3.8 (1.4, 8.8)	2.4 (1.2, 6.4)	3.4 (1.0, 8.6)
**IBDQ total score, median (Q1, Q3)**	141.0 (118.0, 158.0)	130.0 (104.0, 155.0)	125.5 (108.5, 145.0)	130.0 (104.0, 149.0)
**Number of prior failed biologics or tofacitinib, *n* (%)**				
** 0**	64 (70.3%)	150 (61.2%)	22 (78.6%)	87 (60.4%)
** 1**	11 (12.1%)	61 (24.9%)	3 (10.7%)	31 (21.5%)
** ≥2**	16 (17.6%)	34 (13.9%)	3 (10.7%)	26 (18.1%)
**Baseline UC therapy, *n* (%)**				
** Corticosteroids**	32 (35.2%)	92 (37.6%)	8 (28.6%)	57 (39.6%)
** Immunomodulators**	21 (23.1%)	51 (20.8%)	5 (17.9%)	32 (22.2%)
** Aminosalicylates**	70 (76.9%)	187 (76.3%)	19 (67.9%)	108 (75.0%)

Missing values are not included for the calculation of mean, SD, median, Q1, and Q3. Missing records are excluded from the denominator in the calculation of frequency percentages.

Abbreviations: BMI, body mass index; BU, bowel urgency; CRP, C-reactive protein; IBDQ, Inflammatory Bowel Disease Questionnaire; mMS, modified Mayo score; *M*, number of responders/non-responders described in footnotes a and b below; *N*, number of patients; Q, quartile; SC, subcutaneously; SD, standard deviation; UC, ulcerative colitis; UNRS, Urgency Numeric Rating Scale.

a Responder to the combined endpoint of Clinical Remission, BU Remission, and IBDQ Remission. ^b^Non-responder to the combined endpoint of Clinical Remission, BU Remission, and IBDQ Remission.

## Discussion

Treatment goals for UC have advanced from resolution of the traditional symptoms rectal bleeding and frequent stool to the addition of endoscopic healing. Now with a growing emphasis on patient-centric care, treatment goals for UC should also include the resolution of BU and improvement in overall well-being. These newer treatment targets are increasingly recognized as essential for achieving meaningful and comprehensive management of the disease.

We demonstrated in this post-hoc analysis that patients from LUCENT trials who achieved both clinical response or remission and improvements in BU were associated with a better quality of life. Furthermore, we highlighted the impact of mirikizumab in patients with UC, showing substantial improvements in achieving clinical remission, BU remission, and IBDQ remission concurrently compared with placebo. These findings underscore the multidimensional benefits of mirikizumab on aspects of health that matter most to the overall well-being of patients with UC.

The 2019 and 2025 ACG guidelines included the treatment goal of BU remission and recommended that initial treatment of UC focus on restoration of normal bowel frequency and control of the primary symptoms of bleeding and urgency.^12^^,^^13^

This exploratory analysis of patients from the LUCENT-1 and LUCENT-2 trials linked the addition of improvement in BU (CMI or remission) to the achievement of traditional clinical endpoints including clinical response and remission and was significantly associated with greater improvement of quality of life as assessed by IBDQ response and remission. These findings support the importance of assessing both BU and clinical disease activity in clinical practice.^16^

Due to the multifactorial etiologies of UC, BU is likely driven by multiple mechanisms. Thus, improvements in BU may not directly correspond to improvements in other symptoms following treatment. Uncontrolled BU may result in suboptimal patient outcomes, with BU frequently cited as one of the primary reasons for treatment dissatisfaction.^4–6^^,^^17^ Many patients report ongoing BU even when they are classified as in remission based on stool frequency, rectal bleeding, and endoscopic findings.^4^^,^^6^ Approximately 35%-40% of patients with normal stool frequency and minimal rectal bleeding still suffer from BU^11^ which is associated with reduced patients’ quality of life, physical and social activities, travel, and work, as well as increased feelings of stress, anxiety, and social isolation.^5^^,^^17–19^

There is a notable disconnect between patients and health care providers in relation to BU. In the Inflammatory Bowel Disease Global Assessment of Patient and Physician Unmet Needs Survey, BU was frequently reported by patients as a symptom; however, it was not among the top-reported symptoms as perceived by healthcare providers.^20^ In concert with the data presented in this analysis, there is clear evidence to indicate the importance of BU monitoring during UC treatment.

The United States Food and Drug Administration Draft Guidance for the development of patient-reported outcomes^21^ has been updated to include methods for monitoring UC, including the Symptoms and Impacts Questionnaire for UC,^22^ Ulcerative Colitis Patient-Reported Outcomes Signs and Symptoms diary,^23^ and the UNRS.^7^^,^^8^ The UNRS, which assesses the severity of BU and has demonstrated validity and reliability allows health care professionals to examine changes over time. Thus, it was the ideal tool for evaluating BU in the LUCENT-1 and -2 studies.

Schreiber et al. (2024) introduced the concept of “comprehensive disease control (CDC)” for UC, emphasizing a patient-centered approach that extends beyond traditional definitions of clinical remission. CDC incorporates patient-prioritized outcomes and long-term indicators such as endoscopic findings and inflammatory biomarkers.^16^ They proposed that the CDC should combine clinical and patient-reported measures, including rectal bleeding, stool frequency, BU, abdominal pain, extraintestinal manifestations, quality of life, histological activity, biomarker levels, corticosteroid use, fatigue, and sleep disturbance, to support individualized treatment and serve as a meaningful endpoint in both clinical practice and trials.

As supported by studies like LUCENT, CALM, EXTEND, and the STRIDE consensus, combining multiple endpoints enhances overall outcomes.^2^^,^^16^^,^^24–26^ We anticipate this multidimensional approach will be increasingly adopted and further advance patient-centered care.^27^

The randomized withdrawal study design of LUCENT-2 restricted the primary study population to induction responders of LUCENT-1.^11^ Additionally, the observed impact of mirikizumab on multidimensional aspects of disease control in UC merits additional study to further evaluate its comprehensive therapeutic benefits including biomarkers, histology, etc. This analysis was post hoc in nature; therefore, all findings should be interpreted as associative rather than causal. Although we observed strong relationships between BU improvement, clinical outcomes, and quality of life, the study design does not allow determination of whether changes in one outcome directly lead to changes in another. Some analyses pooled treatment arms to evaluate associations, which may obscure treatment-specific effects. The LUCENT-2 randomized withdrawal design also limits interpretation of Week 52 placebo comparisons, as this group does not represent a true parallel placebo cohort. Finally, residual confounding and missing data cannot be fully excluded despite the use of non-responder imputation. These limitations underscore the need for prospective studies specifically designed to test causal pathways between BU, clinical remission, and patient-reported outcomes.

## Conclusion

In this post-hoc analysis of LUCENT trials, mirikizumab is associated with a patient-centric benefit for patients with UC by relieving symptoms, resolving endoscopic inflammation, and enhancing overall patient well-being. Improvement of BU is linked to an additional impact on patients’ quality of life, even among those who achieved clinical remission. A multi-target comprehensive approach in UC is feasible and impacts patient outcomes.

## Supplementary Material

otag051_Supplementary_Data

## Data Availability

Data are available on reasonable request. We will provide access to all individual participant data collected during the trial, after anonymization. Data are available upon request after primary publication acceptance. No expiration date of data requests is currently set once data are made available. Access is provided after a proposal has been approved by an independent review committee identified for this purpose and after receipt of a signed data sharing agreement. Data and documents, including the study protocol, statistical analysis plan, clinical study report, and blank or annotated case report forms, will be provided in a secure data sharing environment. For details on submitting a request, see the instructions provided at www.vivli.org.
