# Supplementary material for: Combined bowel urgency, clinical outcomes, and quality of life improvement in mirikizumab-treated ulcerative colitis: a step toward comprehensive disease management
Source: Crohns Colitis 360. 2026 Jun 10;8(2):otag051. doi: 10.1093/crocol/otag051 (PMC13293077; doi:10.1093/crocol/otag051)
Supplement: otag051_Supplementary_Data [file otag051_supplementary_data.docx]

**Supplemental Table 1: Summary of demographics and other baseline characteristics for mirikizumab- and placebo-treated patients achieving versus not achieving combined clinical remission, BU remission, and IBDQ remission at week 12**

|  | **Combined Endpoint: Clinical Remission, BU Remission, and IBDQ Remission** | | | |
| --- | --- | --- | --- | --- |
|  | **Induction: Week 12**  **Mirikizumab 300mg IV (N=811)** | | **Induction: Week 12**  **Placebo IV (N=276)** | |
|  | **Responders^a^**  **(M=71)** | **Non-responders^b^**  **(M=740)** | **Responders^a^**  **(M=13)** | **Non-responders^b^**  **(M=263)** |
| Age, mean years (SD) | 42.4 (12.5) | 42.8 (14.0) | 38.9 (14.6) | 41.1 (13.4) |
| Male, n (%) | 36 (50.7%) | 458 (61.9%) | 8 (61.5%) | 145 (55.1%) |
| BMI, n (%)  Normal  Underweight  Overweight/Obese/Extreme obese | 47 (66.2%)  3 (4.2%)  21 (29.6%) | 370 (50.0%)  49 (6.6%)  321 (43.4%) | 6 (46.2%)  0  7 (53.8%) | 132 (50.2%)  28 (10.6%)  103 (39.2%) |
| Disease location, n (%)  Left-sided colitis  Pancolitis  Proctitis | 52 (73.2%)  18 (25.4%)  1 (1.4%) | 459 (62.0%)  276 (37.3%)  5 (0.7%) | 8 (61.5%)  5 (38.5%)  0 | 166 (63.4%)  94 (35.9%)  2 (0.8%) |
| mMS, n (%)  [0–6]  [7–9] | 32 (45.1%)  39 (54.9%) | 327 (44.2%)  413 (55.8%) | 8 (61.5%)  5 (38.5%) | 114 (43.5%)  148 (56.5%) |
| Mayo endoscopic subscore (ES), n (%)  Moderate disease (ES=2)  Severe disease (ES=3) | 31 (43.7%)  40 (56.3%) | 232 (31.4%)  508 (68.6%) | 7 (53.8%)  6 (46.2%) | 79 (30.2%)  183 (69.8%) |
| Bowel Urgency Severity (UNRS), mean (SD) | 5.8 (1.8) | 6.5 (1.8) | 6.1 (1.8) | 6.6 (1.9) |
| Faecal calprotectin, µg/g, median (Q1, Q3) | 2094.0  (577.0, 3930.0) | 1565.0  (636.0, 3202.0) | 1063.0  (520.0, 1250.0) | 1511.0  (681.0, 3087.0) |
| CRP, mg/L, median (Q1, Q3) | 3.4  (1.2, 7.7) | 4.3  (1.7, 10.2) | 3.6  (1.4, 12.8) | 4.3  (1.2, 9.9) |
| IBDQ total score, median (Q1, Q3) | 143.5  (114.0, 154.0) | 129.0  (106.0, 152.0) | 147.0  (137.0, 169.0) | 123.0  (101.0, 147.0) |
| Number of prior failed biologics or tofacitinib, n (%) |  |  |  |  |
| 0 | 53 (74.6%) | 414 (55.9%) | 12 (92.3%) | 149 (56.7%) |
| 1 | 8 (11.3%) | 162 (21.9%) | 0 | 63 (24.0%) |
| ≥2 | 10 (14.1%) | 164 (22.2%) | 1 (7.7%) | 51 (19.4%) |
| Baseline UC therapy, n (%)  Corticosteroids  Immunomodulators  Aminosalicylates | 22 (31.0%)  10 (14.1%)  56 (78.9%) | 306 (41.4%)  186 (25.1%)  549 (74.2%) | 8 (61.5%)  6 (46.2%)  10 (76.9%) | 101 (38.4%)  60 (22.8%)  192 (73.0%) |

^a^ Responder to the combined endpoint of Clinical Remission, BU Remission & IBDQ Remission

^b^ Non-responder to the combined endpoint of Clinical Remission, BU Remission & IBDQ Remission

Missing values are not included for the calculation of mean, SD, median, Q1, and Q3.

Missing records are excluded from the denominator in the calculation of frequency percentages.

BMI, body mass index; BU, bowel urgency; CRP, C-reactive protein; IBDQ, Inflammatory Bowel Disease Questionnaire; IV, intravenously; mMS, modified Mayo score; M, number of responders/non-responders described in footnotes a and b below; N, number of patients; Q, quartile; SD, standard deviation; UC, ulcerative colitis; UNRS, Urgency Numeric Rating Scale

**Supplemental Figure 1:** IBDQ response and remission with (A) or without (B) BU CMI and with (C) or without (D) BU remission at weeks 12 and 52 (NRI). The addition of BU CMI or BU remission to clinical response/remission is associated with higher IBDQ response and remission at weeks 12 and 52 in both mirikizumab-treated and placebo subgroups.

*P≤.05; **P≤.01 ****P*≤.001; *****P*≤.0001, with (+) BU remission vs without (-) BU remission, chi-square test of association. The difference between with and without BU remission was not adjusted for covariates and was not continuity corrected.

BU remission: UNRS score of 0 or 1. BU CMI: Change in baseline in UNRS ≥3 in patients with UNRS ≥3 at induction baseline.

BU, Bowel Urgency; CMI, clinically meaningful improvement; IBDQ, Inflammatory Bowel Disease Questionnaire; NRI, non-responder imputation; NRS, Numeric Rating Scale; N, number of patients in the denominator; UNRS, Urgency Numeric Rating Scale; W, week.
